# Supplementary material for: Prevalence and Risk Factors for Hyposalivation and Xerostomia in Childhood Cancer Survivors Following Different Treatment Modalities—A Dutch Childhood Cancer Survivor Study Late Effects 2 Clinical Study (DCCSS LATER 2)
Source: Cancers (Basel). 2022 Jul 11;14(14):3379. doi: 10.3390/cancers14143379 (PMC9320024; doi:10.3390/cancers14143379)
Supplement: Supplementary file 1 [file cancers-14-03379-s001.zip › cancers-1774319-supplementary.pdf]

Prevalence and Risk Factors for Hyposalivation and Xerostomia in Childhood Cancer Survivors Following Different Treatment Modalities—A Dutch Childhood Cancer Survivor Study Late Effects 2 Clinical Study (DCCSS LATER 2)

Juliette Stolze [1,2,3,*], Jop C. Teepen [1], Judith E. Raber-Durlacher [3,4], Jacqueline J. Loonen [5], Judith L. Kok [1], Wim J. E. Tissing [1,6], Andrica C. H. de Vries [1,7], Sebastian J. C. M. M. Neggers [8], Eline van Dulmen-den Broeder [9], Marry M. van den Heuvel-Eibrink [1], Helena J. H. van der Pal [1], A. Birgitta Versluys [1], Margriet van der Heiden-van der Loo [1], Marloes Louwerens [10], Leontien C. M. Kremer [1,11,12], Henk S. Brand [2] and Dorine Bresters [1] on behalf of the DCCSS LATER Study Group

1 Princess Máxima Center for Pediatric Oncology, 3584 CS Utrecht, The Netherlands; j.c.teepen@prinsesmaximacentrum.nl (J.C.T.); j.l.kok-5@prinsesmaximacentrum.nl (J.L.K.); W.J.E.Tissing@prinsesmaximacentrum.nl (W.J.E.T.); A.C.H.deVries-15@prinsesmaximacentrum.nl (A.C.H.d.V.); m.m.vandenheuvel-eibrink@prinsesmaximacentrum.nl (M.M.v.d.H.-E.); h.j.h.vanderpal@prinsesmaximacentrum.nl (H.J.H.v.d.P.); a.b.versluijs@prinsesmaximacentrum.nl (A. B.V.); m.vanderheiden@prinsesmaximacentrum.nl (M.v.d.H.-v.d.L.); l.c.m.kremer@prinsesmaximacentrum.nl (L.C.M.K.); d.bresters@prinsesmaximacentrum.nl (D.B.)
2 Department of Oral Biochemistry, Academic Center for Dentistry Amsterdam (ACTA), 1081 LA Amsterdam, The Netherlands; h.brand@acta.nl (H.S.B.)
3 Department of Oral Medicine, Academic Center for Dentistry Amsterdam (ACTA), 1081 LA Amsterdam, The Netherlands; judith@raber.nl (J.E.R.-D.)
4 Department of Oral and Maxillofacial Surgery, Amsterdam University Medical Center (UMC), Location AMC, 1105 AZ Amsterdam, The Netherlands
5 Radboud University Medical Center, 6525 GA Nijmegen, The Netherlands; Jacqueline.loonen@radboudumc.nl (J.J.L.)
6 Department of Pediatric Oncology, Beatrix Children's Clinic, University Medical Center Groningen,
9713 GZ Groningen, The Netherlands
7 Department of Pediatric Oncology, Sophia Children's Hospital, Erasmus Medical Center,
3015 GD Rotterdam, The Netherlands
8 Department of Internal Medicine, Section Endocrinology, Erasmus Medical Center, 3015 GD Rotterdam, The Netherlands; s.neggers@erasmusmc.nl (S.J.C.M.M.N.)
9 Emma Children's Hospital, Amsterdam UMC, Location VUmc, 1105 AZ Amsterdam, The Netherlands; eline.vandulmen-denbroeder@vumc.nl (E.v.D.-d.B.)
10 Department of Internal Medicine/Endocrinology, Leiden University Medical Center, 2333 ZA Leiden, The Netherlands; m.louwerens@lumc.nl (M.L.)
11 Wilhelmina Children's Hospital, University Medical Center Utrecht, 3584 EA Utrecht, The Netherlands
12 Emma Children's Hospital, Amsterdam UMC, Location AMC, 1105 AZ Amsterdam, The Netherlands
* Correspondence: jstolzetandarts@gmail.com (J.S.); Tel.: +31-(0)88 9725192

SUPPLEMENTARY MATERIAL

Table S1. Type of cancer.

Type of cancer	Number (%)	Group 1: No H&N RT	Group 2: H&N RT
Leukemias, myeloproliferative diseases and myelodysplastic diseases	169 (57.9)	111 (56.1)	58 (61.7)
Lymphoid leukemias	135 (46.2)	91	44
Acute myeloid leukemias	19 (6.5)	11	8
Chronic myeloproliferative diseases	4 (1.4)	1	3
Myelodysplastic syndrome and other myeloproliferative diseases	10 (3.4)	7	3
Unspecified and other specified leukemias	1 (0.3)	1	0
Lymphomas and reticuloendothelial neoplasms	47 (16.1)	36 (18.2)	11 (11.7)
Hodgkin lymphomas	20 (6.8)	13	7
Non-Hodgkin lymphomas (excluding Burkitt lymphoma)	15 (5.1)	13	2
Burkitt lymphoma	6 (2.1)	4	2
Langerhans cell histiocytosis	6 (2.1)	6	0
CNS and miscellaneous intracranial and intraspinal neoplasms	19 (6.5)	1 (0.5)	18 (19.1)
Ependymomas and choroid plexus tumor	5 (1.7)	0	5
Astrocytomas	4 (1.4)	0	4
Intracranial and intraspinal embryonal tumors	9 (3.1)	1	8
Other gliomas	1 (0.3)	0	1
Neuroblastoma and other peripheral nervous cell tumors	4 (1.4)	4 (2.0)	0 (0.0)
Neuroblastoma and ganglioneuroblastoma	4 (1.4)	4	0
Renal tumors	20 (6.8)	20 (10.1)	0 (0.0)
Nephroblastoma and other nonepithelial renal tumors	20 (6.8)	20	0
Hepatic tumors	1 (0.3)	1 (0.5)	0 (0.0)
Hepatoblastoma	1 (0.3)	1	0
Bone tumors	16 (5.5)	15 (7.6)	1 (1.1)
Osteosarcomas	9 (3.1)	8	1
Chondrosarcomas	1 (0.3)	1	0
Ewing tumor and related sarcomas of bone	6 (2.1)	6	0
Soft tissue and other extraosseous sarcomas	11 (3.8)	7 (3.5)	4 (4.3)
Rhabdomyosarcomas	7 (2.4)	4	3
Fibrosarcomas, peripheral nerve sheath tumors, and other fibromatous neoplasms	2 (0.7)	2	0
Other specified soft tissue sarcomas	1 (0.3)	1	0
Unspecified soft tissue sarcomas	1 (0.3)	0	1
Germ cell tumors, trophoblastic tumors, and neoplasms of gonads	4 (1.4)	3 (1.5)	1 (1.1)
Intracranial and intraspinal germ cell tumors	1 (0.3)	0	1
Malignant extracranial and extragonadal germ cell tumors	3 (1.0)	3	0
Other malignant epithelial neoplasms and malignant melanomas	1 (0.3)	0 (0.0)	1 (1.1)
Other and unspecified carcinomas	1 (0.3)	0	1
Total	292 (100.0)	198 (100.0)	94 (100.0)

Numbers do not always add up to 100% because of rounding.

Table S2. Univariable Poisson regression analyses between different potential risk factors and hyposalivation measured by UWS, SWS and xerostomia based on the XI-questionnaire.

Variable	Hyposalivation, UWS (<0.2mL/min)	Hyposalivation, SWS (<0.7mL/min)	Xerostomia
Gender			
Male	ref	ref	ref
Female	OR 1.526 (1.072-2.172) p 0.019	OR 1.547 (1.086-2.203) p 0.016	OR 1.389 (0.625-3.087) p 0.420
Age at diagnosis (per 1 year increase)	OR 1.016 (0.979-1.055) p 0.392	OR 0.990 (0.951-1.031) p 0.629	OR 1.050 (0.957-1.152) p 0.304
Time since diagnosis (per 10 year increase)	OR 1.599 (1.331-1.922) p<0.001	OR 1.130 (0.907-1.408) p 0.277	OR 0.906 (0.559-1.470) p 0.691
Age at enrollment of the study (per 10 year increase)	OR 1.479 (1.255-1.742) p<0.001	OR 1.070 (0.877-1.305) p 0.504	OR 1.049 (0.705-1.562) p 0.812
Radiotherapy dose to salivary glands			
0 Gy	ref	ref	ref
>0 and ≤12 Gy	OR 1.484 (0.862-2.552) p 0.154	OR 1.499 (0.871-2.580) p 0.144	OR 1.500 (0.467-4.823) p 0.496
>12 and ≤34 Gy	OR 1.929 (1.265-2.941) p 0.002	OR 1.705 (1.075-2.704) p 0.023	OR 0.885 (0.213-3.669) p 0.866
>34 Gy	OR 2.057 (1.209-3.499) p 0.008	OR 2.339 (1.449-3.774) p 0.001	OR 1.437 (0.358-5.769) p 0.609
per 10 Gy increase	OR 1.170 (1.095-1.250) p<0.001	OR 1.155 (1.049-1.272) p 0.003	OR 1.055 (0.838-1.329) p 0.648
Chemotherapy			
Vinca alkaloids	OR 0.926 (0.588-1.457) p 0.738	OR 0.564 (0.393-0.809) p 0.002	-*
Anthracyclines	OR 0.891 (0.624-1.272) p 0.526	OR 0.641 (0.455-0.904) p 0.011	OR 1.208 (0.513-2.844) p 0.665
Alkylating agents	OR 0.848 (0.591-1.216) p 0.369	OR 0.666 (0.471-0.941) p 0.021	OR 2.221 (0.778-6.338) p 0.136
Epipodophyllotoxins	OR 0.777 (0.513-1.178) p 0.235	OR 1.053 (0.721-1.537) p 0.788	OR 1.358 (0.597-3.090) p 0.465
Platinum Compounds	OR 0.763 (0.392-1.485) p 0.426	OR 0.890 (0.482-1.644) p 0.710	OR 1.769 (0.648-4.827) p 0.265
Antimetabolites	OR 0.992 (0.682-1.444) p 0.967	OR 1.307 (0.867-1.970) p 0.201	OR 1.551 (0.595-4.043) p 0.369
TBI	OR 0.885 (0.528-1.482), p 0.642	OR 1.032 (0.605-1.759) p 0.908	OR 1.543 (0.419-5.684) p 0.514
Polypharmacy			
0-3 medications	ref	ref	ref
>3 medications	OR 1.423 (0.919-2.206) p 0.114	OR 0.855 (0.475-1.539) p 0.602	OR 1.951 (0.777-4.897) p 0.155
Number of medications (continuous)	OR 1.114 (1.046-1.186) p 0.001	OR 1.044 (0.961-1.134) p 0.308	OR 1.228 (1.074-1.404) p 0.003

*Gy: gray, (ref): reference category. * Analysis was not possible due to zero participants with xerostomia in the non vinca alkaloids category.*
